# Lag time from calving to first colostrum harvest in Holstein dairy cows: Association with colostral immunoglobulin G, volume, and dry matter

**DOI:** 10.3168/jdsc.2025-0770

**Published:** 2025-07-01

**Authors:** Sabine Mann, Madeleine Spellman, Grace Frederick, Rupert Bruckmaier, Haritha Somula, Matthias Wieland

**Affiliations:** 1 Department of Population Medicine and Diagnostic Sciences, College of Veterinary Medicine, Cornell University, Ithaca, NY 14853; 2 Veterinary Physiology, Vetsuisse Faculty, University of Bern, Bern CH-3001, Switzerland

## Abstract

The objective of this observational study was to describe the association between lag time from calving to first milking for colostrum harvest. Colostrum samples from primiparous and multiparous Holstein cows (n = 640) from a single herd milking approximately 5,200 cows 3 times daily in a 100-stall rotary parlor were used in this prospective cohort study. Calves were removed immediately after calving and not allowed to suckle their dam. Colostrum was harvested twice daily at 0930 and 1730 h and stored in a colostrum bank, from which all newborn calves were fed within 2 h after birth. Only cows milked at 0930 h were eligible for enrollment. Time of calving was recorded and time lag to the morning milking at 0930 h was calculated. Total yield of the colostrum was determined by weight. Colostrum samples were analyzed for IgG concentration by radial immunodiffusion and DM content by oven drying for 20 h at 100°C. Lag time in this cohort had a median of 10 h, ranged from 0 to 18 h, and was divided into 3 h increments to assess associations with outcomes of interest. Additionally, data were explored using segmented regression analyses. Total IgG yield was calculated from yield and IgG concentration. The association of colostral yield, IgG concentration, DM, as well as total IgG yield was analyzed for the different lag time groups using mixed effects linear regression. All lag time groups were compared with the control level 0 to 3 h using Dunnett's test. A total of 203 (31.7%), 149 (23.3%), 113 (17.7%), and 175 (27.3%) animals were in lactations 1, 2, 3, and ≥4, respectively. Cows were distributed with 78 (12.2%) in group 0 to 3 h, 116 (18.1%) in group >3 to 6 h, 96 (15%) in group >6 to 9 h, 123 (19.2%) in group >9 to 12 h, 110 (17.2%) in group >12 to 15 h, and 117 (18.3%) in the >15 to 18 h group. When lag time exceeded 9 h, the concentration of IgG and DM percentage were lower than in the 0 to 3 h control level. Colostrum yield was higher than the control level when cows were milked for the first time >15 h after calving. Segmented linear regression identified a breakpoint at 8.3 h for a decrease in IgG concentration and DM, as well as 5.7 h for an increase in yield. Lag time was not observed to be associated with total IgG yield in either model. After 9 h, a dilution effect was observed as demonstrated by a drop in IgG concentration and DM with no associated change in total IgG yield.

## Highlights

• We investigated associations of lag time from calving to first milking of colostrum.
• Delaying first milking for >9 hours was associated with lower IgG and DM.
• No change in total IgG suggested a dilution effect with increased lag time.

## Graphical Abstract

**Figure:**
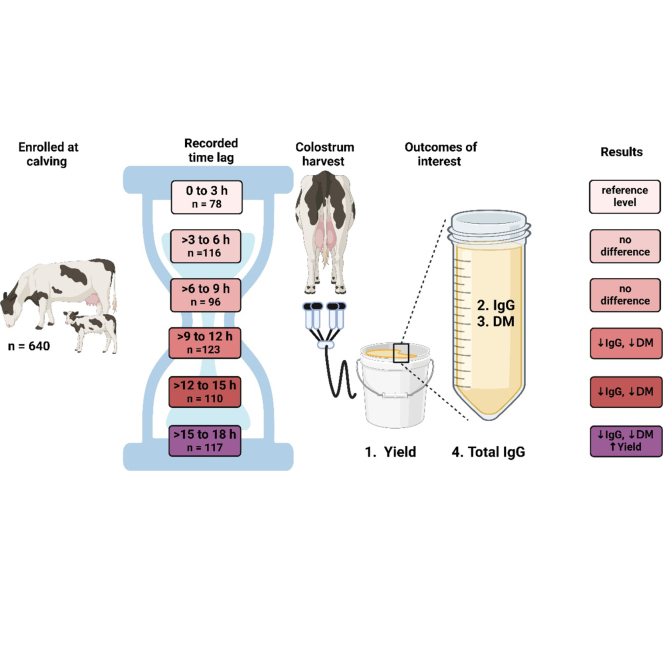
**Summary:** First-milking colostrum samples (n = 640) were collected from Holstein cows up to 18 hours after calving. Colostrum yield was recorded, and colostrum samples were analyzed for immunoglobulin G (IgG) concentration and dry matter (DM). Total IgG yield was calculated from colostrum yield and concentration for each sample. A lag time of over 9 hours from calving to milking was associated with a decrease in colostral IgG concentration and DM. Total IgG yield did not change over time. When stored, high-quality colostrum is available to feed the newborn calf immediately after birth, first milking of colostrum can be delayed up to 9 hours without significant reduction in IgG concentration. A dilution effect was measurable after 9 hours.

Dairy producers are advised to feed an adequate amount of high-quality colostrum to calves in the first hours of life to maximize transfer of passive immunity and other benefits attributed to colostrum feeding (Godden et al., 2019). Specifically, efficiency of uptake of IgG rapidly decreases in the first 12 h after birth due to rapid gut closure as recently reviewed by Lopez and Heinrichs (2022). The need to feed newborn calves quickly after birth is undebated and critically important for the newborn, but colostrum harvest is often managed independently of the first feeding on farms where banked colostrum is used. As an example, 95% (18/19) of dairy producers in a recent survey in New York State fed newborn calves within 2 h after birth, but only 16% (3/19) harvested colostrum in the same timeframe, and only 11% (2/19) fed calves their own dam's colostrum (Westhoff et al., 2023). Robbers et al. (2021) found in a survey of producers in the Netherlands that a similarly large proportion of producers (84%, 87/104) fed calves colostrum within 2 h of birth; however, on most farms (96%, 100/104) calves received their own dam's colostrum. Thus, although some producers will milk colostrum within a few hours after calving, others will allow a greater variability in lag time from calving to first milking depending on their chosen management strategy and use of a colostrum bank. To harvest colostrum, postpartum cows are either milked individually by hand, by bucket or in a parlor, either immediately or at the next scheduled milking time in a group (Westhoff et al., 2023).

The available literature suggests that colostrum quality, defined as IgG concentration, will continuously decline after parturition and with an increasing lag time between calving and milking. Morin et al. (2010) described a 3.7% decline in IgG concentration with every hour after calving in 56 multiparous cows from a single herd. Moore et al. (2005) experimentally tested the effect of time lag in a prospective study where each of 4 quarters of 13 Holstein cows were randomized to be milked at 2, 6, 10, or 14 h after calving. The authors described a linear relationship such that quarters milked at 6, 10, and 14 h after calving had significantly and increasingly less IgG concentration although quarter-level colostrum yield was similar; however, results should be interpreted with caution as colostrum composition in the nonmilked quarters could have been influenced by milking of the other quarters. Based on these results, recommendations have traditionally been made to prevent a significant time lag before first milking to preserve the quality of colostrum (Moore et al., 2005; Morin et al., 2010). However, although newer data suggest that there is a decline in IgG concentration with increasing lag time from calving to colostrum collection, the onset of such a decline is not consistent across studies. Quigley et al. (2013) visually observed a decline in IgG concentration after approximately 8 h in a sample from 173 Holstein cows in the United States. This decline was similar to that in IgG concentration after 9 h described in a sample of 704 seasonally calving pastured cows from 4 herds in Ireland (Conneely et al., 2013), but different from a decline already detected at 3 h, with a second significant decline at 12 h in colostrum samples from 458 cows of 13 different breeds in Switzerland and Germany (Kessler et al., 2020a). Reschke et al. (2017) included 377 colostrum samples from cows of different breeds on 141 Swiss dairy farms and categorized time lag as <2, 2 to ≤6, or >6 h and found that samples collected after 6 h had a significantly higher proportion of poor quality (IgG <50 g/L).

Additionally, it is currently unclear if the reduction in colostral IgG over time is due to a dilution effect or loss of IgG from the mammary gland. Both possibilities have been discussed in previous works, with certain authors favoring the theory that there is a simple dilution effect due to an increase in overall yield at first milking (Morin et al., 2010; Kessler et al., 2020a), whereas others hypothesize that IgG could be taken back up by the mammary gland into tissues or circulation (Moore et al., 2005; Conneely et al., 2013).

Thus, it is important to investigate if lag time for colostrum milking is associated with an increase in DM percentage in cow colostrum with time so that producers can understand the changes associated with choosing to delay colostrum milking in their daily routine. Therefore, our objectives were to investigate the association between lag time from calving with IgG concentration as well as the change in DM and colostrum volume in an observational study using a well-characterized sample set. We hypothesized that lag time is associated with colostrum quality, defined as IgG concentration.

This study was conducted as a prospective cohort study. Animal management and colostrum harvest procedures were as previously described in detail (Mann et al., 2024). In brief, Holstein cattle from a single commercial dairy farm in New York were enrolled in this study between July and October 2023. Close-up pens were walked by farm personnel hourly, and cows were moved to a bedded straw pack (3.8 m × 9.5 m) in a just-in-time manner at the first signs of stage II labor (Carrier et al., 2006). Farm personnel attended all births, removed the calf immediately from the dam, and recorded the time of parturition. All calves were fed colostrum within 2 h after birth from the farm's colostrum bank. According to the farm protocol, cows were milked for the first time after calving in a 100-stall rotary parlor (RP3100HD, DeLaval International AB) twice daily at 0930 and 1730 h. Only animals milked for the first time during the morning milking (0930 h) with a calving during the preceding 24 h were eligible for enrollment. For a different study objective, and following a randomized block design, animals were either given 0, 20, or 40 IU oxytocin (20 IU/mL, VetOne) intramuscularly with a 20-G 2.5-cm-long needle approximately 45 s before milking unit attachment.

Colostrum was milked into translucent polypropylene buckets and the weight was recorded immediately after milking as described previously (Mann et al., 2024). Colostrum was carefully mixed, and composite samples were collected for transport to the laboratory and storage at −20°C until further analysis. Whole colostrum was thawed and IgG concentration was determined by radial immunodiffusion, and DM percentage was calculated by oven drying as previously described (Mann et al., 2024). Total IgG yield was estimated by multiplication of the IgG concentration by the colostrum weight and simplifying the weight to volume ratio to 1, instead of applying the specific gravity of colostrum of approximately 1.05 (Parrish et al., 1950; Quigley et al., 1994).

Sample size was not determined for this study specifically, instead we used all samples available (n = 640) from the parent study described in Mann et al. (2024). This sample size was deemed sufficient to detect at least a medium effect size (≥0.20) for a difference between the 6 groups with greater than 95% power (0.957), an α probability of 0.05, and 4 covariates (GPower 3.1.9.7, Faul et al., 2007). The lag time from calving to first milking was divided into 6 groups of 3 h increments (0 to 3, >3 to 6, >6 to 9, >9 to 12, >12 to 15, >15 to 18 h) similar to Conneely et al. (2013). Lactation number was grouped into lactation 1, 2, 3, or ≥4 and forced into all models. Colostrum samples were dichotomized into those ≤50 g/L (poor quality) and >50 g/L (acceptable quality). The association of this parameter with lag time group was explored using Fisher's exact test. For analysis, group differences between categorized variables (lactation, oxytocin treatment, poor or acceptable quality) were examined using chi-squared tests, and models for the outcome of colostral IgG concentration, yield, DM, and total IgG were performed as mixed effects linear regression in JMP Pro (v. 16.0, SAS Institute Inc.). All regression models included the fixed effect of lag time, the potential confounder of oxytocin treatment group (0, 20, or 40 IU), and the random effect of enrollment block. Additionally, all models were controlled for the potential confounding effect of lactation group as a fixed effect. For all models, the interaction between lag time group and parity (primiparous vs. multiparous, replacing the effect of lactation group) was also tested to investigate if the association of lag time group with the outcomes of interest differed between heifers and cows. The regression model assumptions of normality and homoscedasticity of the residuals were visually assessed. To test the effect of lag time on our outcomes of interest, each lag time group was compared with the shortest lag time group (0 to 3 h) using Dunnett's test to adjust for multiple comparisons. Dunnett's test provides correction of hypothesis testing results when performing multiple pairwise comparisons between each category of interest and a chosen control level (Ludbrook, 1998). Data at each time point are presented as the LSM and 95% CI. Significance was declared when *P* < 0.05 in Dunnett's test.

Additionally, for all outcomes of interest with a linear, quadratic, or cubic relationship with lag time as a continuous variable in the mixed model as specified previously (IgG concentration, yield, DM), segmented regression analyses were subsequently performed in R statistical software (version 4.1.1., R Core Team, 2023). First, we fitted a linear regression model using the lm() function in R, including lag time, lactation number, and treatment as fixed effects. To identify the structural change in the relationships between lag time and the outcome variables IgG concentration, yield, and DM, we fit a segmented linear regression model with 1 breakpoint in lag time with the ‘segmented’ package (Muggeo, 2003). The initial breakpoint estimate was seeded using the median lag time value. The segmented model was estimated using the segmented() function with lag time as the segmented variable. The identified breakpoints were then extracted from the model and used to graph the segmental linear regression for the raw data in GraphPad Prism (v.10.3.1 for Windows, GraphPad Software Inc.).

The median (range) time to first milking in this sample set was 9.9 (0 to 18) h. Animal distribution in the lag time groups is shown in Table 1. Lactation groups were equally distributed in the lag time groups (*P* = 0.40) with 203 (31.7%), 149 (23.3%), 113 (17.7%), and 175 (27.3%) animals in lactations 1, 2, 3, and ≥4, respectively. Oxytocin treatment assignment was not different between lag time categories (*P* = 0.14) but was retained in all models as a possible confounder, regardless of model *P*-value (IgG model, *P* = 0.22; yield model, *P* = 0.02; DM model, *P* = 0.38; and total IgG model, *P* = 0.48). Descriptive analysis of colostral IgG concentration, yield, DM, and total IgG by lag time group is shown in Table 1. We found that lag time category was significantly associated with colostral IgG concentration (*P* < 0.0001), yield (*P* < 0.0001), and DM (*P* < 0.0001), whereas lag time was not observed to be associated with total IgG yield (*P* = 0.25; Figure 1). We did not find an interaction between lag time group and parity (1 vs. ≥2) for any of the aforementioned outcomes (*P* ≥ 0.10). Out of all samples, 71 (11.1%) had an IgG concentration of ≤50 g/L. The proportion of these low-quality samples increased from 0%, 6.9%, 3.1%, 9.9%, 11.8%, to 30.2% of samples in the 0 to 3, >3 to 6, >6 to 9, >9 to 12, >12 to 15, and >15 to 18 h groups, respectively (*P* < 0.001).

**Table 1:** Means (95% CI) of descriptive data for colostrum composite samples of 640 Holstein cows milked at different intervals from calving to first milking for colostrum harvest

Colostrum parameter	0 to 3 h (n = 78)	>3 to 6 h (n = 116)	>6 to 9 h (n = 96)	>9 to 12 h (n = 123)	>12 to 15 h (n = 110)	>15 to 18 h (n = 117)
IgG, g/L	127	115	120	105	93	78
	(119 to 137)	(107 to 124)	(110 to 131)	(96 to 114)	(85 to 101)	(71 to 87)
Yield, kg	4.9	4.6	4.8	4.9	5.8	6.6
	(4.3 to 5.6)	(4.0 to 5.2)	(4.2 to 5.4)	(4.4 to 5.5)	(5.2 to 6.5)	(5.9 to 7.2)
DM, %	25.6	24.9	25.2	23.9	22.5	21.4
	(24.7 to 26.6)	(24.3 to 25.6)	(24.3 to 26.1)	(23.2 to 24.6)	(21.8 to 23.1)	(20.7 to 22.0)
Total IgG, g	532	482	541	495	510	532
	(528 to 700)	(422 to 540)	(475 to 608)	(430 to 561)	(442 to 580)	(438 to 625)

**Figure 1:**
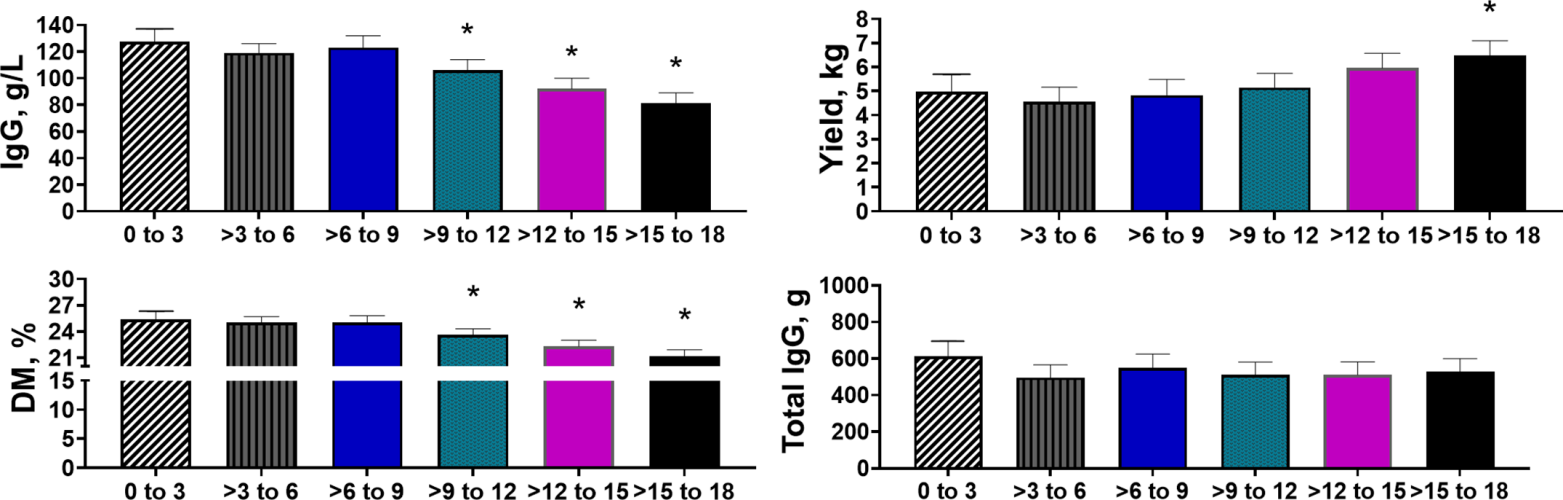
Association of time from calving to harvest (h) as a categorical variable on colostral IgG concentration, yield, DM, and total IgG of composite samples from 640 Holstein cows. Data are presented as LSM and 95% CI. * Denotes differences in lag time category from the control group (0 to 3 h) at a level of *P* < 0.05 in Dunnett's testing. The numbers of animals were 78, 116, 96, 123, 110, and 117 in the 0 to 3, >3 to 6, >6 to 9, >9 to 12, >12 to 15, and >15 to 18 h groups, respectively. Data analysis was performed using mixed effects ANOVA with fixed effects of lag time group, treatment (oxytocin) group, lactation group, and the random effect of enrollment block.

Segmented regression analyses were performed for the association of lag time as a continuous variable with the outcomes IgG concentration, yield, and DM of colostrum, whereas no linear, quadratic, or cubic relationship was identified for the relationship between lag time and total IgG. The identified breakpoints were a decrease in IgG concentration at 8.33 h, a decrease in DM percentage at 8.33 h, and an increase in yield at 5.67 h, respectively (Figure 2).

**Figure 2:**
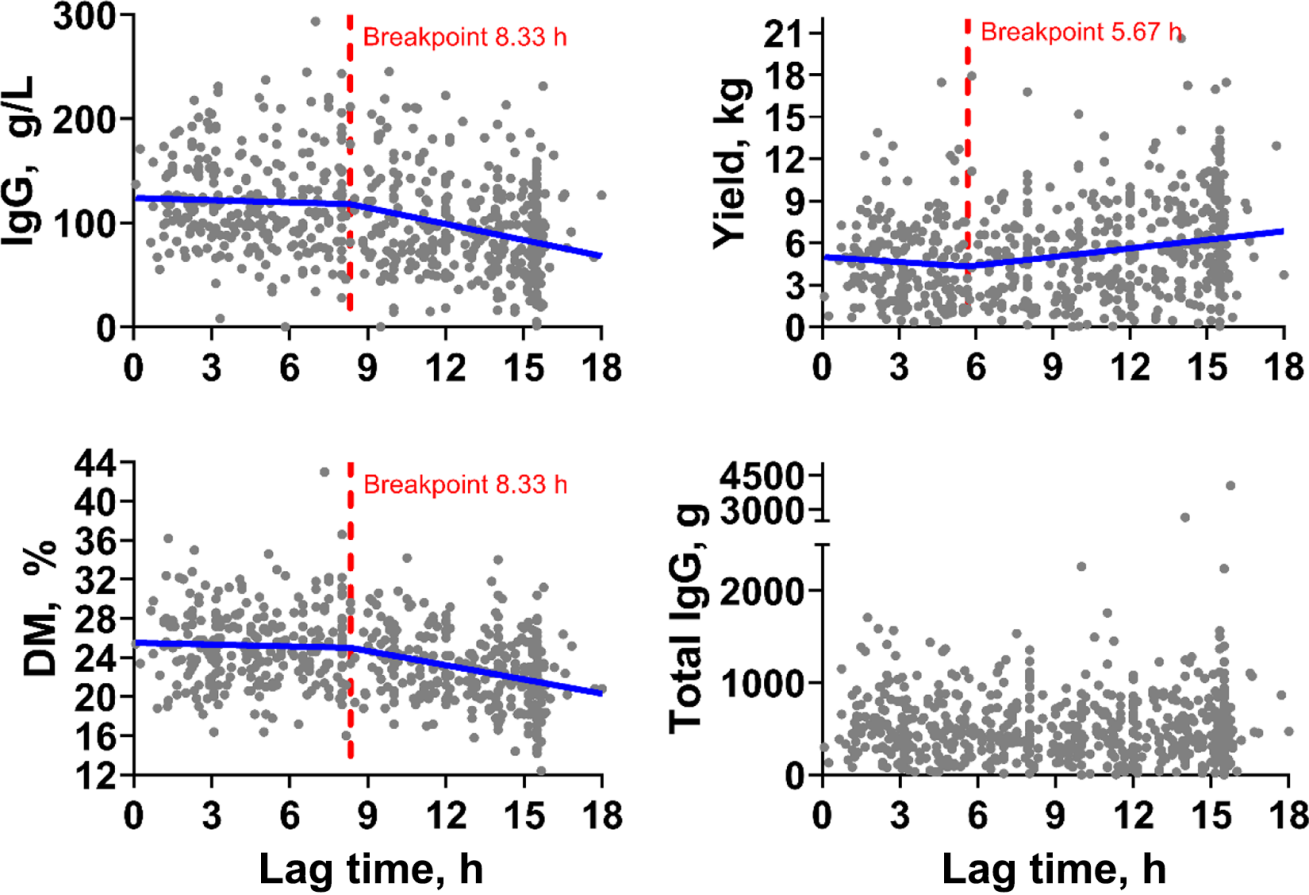
The relationship between lag time (h) modeled as a continuous variable and IgG concentration, yield, DM percentage, and total IgG of colostrum samples from 640 Holstein dairy cows. Raw data points were graphed and breakpoints derived from segmented regression analysis (R statistical software) plotted in GraphPad Prism for IgG concentration, yield, and DM percentage, whereas no linear, quadratic, or cubic relationship existed between lag time and total IgG.

The association of time lag and colostral IgG concentration and yield from observational data has often been modeled assuming a linear (Sutter et al., 2019; Kessler et al., 2020b) or log-linear relationship (Morin et al., 2010). Others have explored the possible relationship either visually (Quigley et al., 2013), or by categorizing time lag instead of assuming a linear relationship (Conneely et al., 2013; Reschke et al., 2017; Kessler et al., 2020a). Whereas the former category of studies suggests a decline in IgG concentration early after calving and with each increasing hour, the latter category, which includes our current study, suggest that changes in colostrum IgG concentration are not measurable until 9 h after calving. While transformation of continuous data into categories often carries the risk of a lack of statistical power (Fedorov et al., 2009) and possible bias due to investigator-driven choice of cut-points, we chose to use a categorization of lag time due to the advantage of interpretability as well as not having to fulfill the statistical assumption of a linear relationship between dependent and independent variables, which was not clearly demonstrated when examining our data. To extend our investigation, continuously modeled data were also explored using a segmented linear regression analysis to allow us to fit piecewise regression terms and identify a breakpoint in our data. Although the breakpoints for IgG concentration and DM changes agreed well between our 2 analyses models, a change in yield was shown sooner after calving in the segmented regression, which could have been due to the increase in power due to the use of continuous data in light of the large variability in yield between cows.

The implications of clarifying the relationship between lag time from calving to first milking and the decrease in colostral IgG concentration should not be underestimated as dairy producers make decisions based on the scientific data presented to them. This allows them to allocate resources appropriately, for example, personnel needed for colostrum harvest. It should be noted that although a time lag from calving to colostrum harvest is acceptable for colostrum quality based on our data, newborn calves should be fed within only a few hours after birth (Lopez and Heinrichs, 2022) to maximize transfer of passive immunity and the benefit of all biologically active components of colostrum, as well as its nutrients. Fischer et al. (2018) found apparent efficiency of absorption (**AEA**) of IgG to be decreased at 6 h (35.6%) and 12 h (35.1%) compared with 45 min (51.8%) after birth. The AEA further declines and reaches only a meager 11.5% at 24 h after birth, as shown by Matte et al. (1982). If colostrum is harvested 3 times per day, most fresh cows are likely to be milked within 8 h after parturition. Our data lend support to this management strategy in terms of preserving IgG concentration of colostrum. However, if colostrum is not harvested shortly after calving, then newborn calves need to be fed banked colostrum to satisfy the critical and time-sensitive need of the neonate for colostrum.

We explored the potential reason for the eventual decline in IgG concentration by measuring both colostral DM percentage and total IgG yield. Changes in DM content serve as indication of a possible dilution effect, whereas total IgG yield is an indicator of overall change (i.e., a potential loss) of colostral IgG mass. Conneely et al. (2013) supported the suggestion by Moore et al. (2005) who disputed the hypothesis that the decrease in IgG concentration is due to dilution alone and instead proposed that colostral IgG could diffuse out of the mammary gland into circulation. Our data support a dilution effect as an underlying reason for the decrease in IgG concentration after 9 h. This is supported by the concurrent DM decline, as well as the absence of a change in total IgG between the lag time groups. A significant increase in colostrum yield was not documented until after 15 h, which is likely due to the large variability in colostrum yield between cows, requiring a substantial biological effect to detect statistical differences. Regardless of the fact that IgG does not appear to be lost from colostrum, newborn calves should receive a volume of colostrum adequate for their birth BW (approximately 10%) in a single feeding, and it is important to feed colostrum at the maximal IgG concentration possible.

We acknowledge our study limitations. We conducted this investigation on a single dairy farm in New York. As a result, the findings are most applicable to dairy operations with similar management practices in this region. The external validity of the study remains limited until the results are replicated in different systems and geographic locations in appropriately large sample sets that include heifers and cows as did our study. Additionally, colostrum production experiences seasonal changes (Westhoff et al., 2023), and data collection was restricted to the summer and fall months, excluding observations from spring and winter. To better understand the relationship between lag time from calving to first colostrum harvest, future research should span an entire year to evaluate potential interactions between time lag and seasonal effects on colostrum yield and quality. Our study only focused on IgG concentration, yield, and DM of colostrum in a convenience sample, but other colostral components such as growth factors and other biologically active components were not investigated and might experience changes in abundance in a similar or different manner compared with the measured outcomes in this study. Last, our data are derived from an observational study, and thus cause-effect relationships should be interpreted with caution. An experiment with assignment of primi- and multiparous cows to different lag time groups for first milking in a randomized controlled study would provide stronger evidence.

In conclusion, when high-quality colostrum is available to feed newborn calves immediately after birth, first milking of colostrum can be delayed for up to 9 h without a significant reduction in IgG concentration. A decline in percentage of DM was measurable after 9 h, but IgG yield did not differ over time. We conclude from these data that IgG remained in the mammary gland but was diluted measurably after 9 h.
